# The potential impact of carboxylic-functionalized multi-walled carbon nanotubes on trypsin: A Comprehensive spectroscopic and molecular dynamics simulation study

**DOI:** 10.1371/journal.pone.0198519

**Published:** 2018-06-01

**Authors:** Maryam Noordadi, Faramarz Mehrnejad, Reza H. Sajedi, Majid Jafari, Bijan Ranjbar

**Affiliations:** 1 Department of Life Science Engineering, Faculty of New Sciences & Technologies, University of Tehran, Tehran, Iran; 2 Department of Biochemistry, Faculty of Biological sciences, Tarbiat Modares University, Tehran, Iran; 3 Department of Biophysics, Faculty of Biological sciences, Tarbiat Modares University, Tehran, Iran; US Naval Research Laboratory, UNITED STATES

## Abstract

In this study, we report a detailed experimental, binding free energy calculation and molecular dynamics (MD) simulation investigation of the interactions of carboxylic-functionalized multi-walled carbon nanotubes (COOH-f-MWCNTs) with porcine trypsin (pTry). The enzyme exhibits decreased thermostability at 330K in the presence of COOH-f-MWCNTs. Furthermore, the activity of pTry also decreases in the presence of COOH-f-MWCNTs. The restricted diffusion of the substrate to the active site of the enzyme was observed in the experiment. The MD simulation analysis suggested that this could be because of the blocking of the S1 pocket of pTry, which plays a vital role in the substrate selectivity. The intrinsic fluorescence of pTry is quenched with increase in the COOH-f-MWCNTs concentration. Circular dichroism (CD) and UV–visible absorption spectroscopies indicate the ability of COOH-f-MWCNTs to experience conformational change in the native structure of the enzyme. The binding free energy calculations also show that electrostatics, π-cation, and π-π stacking interactions play important roles in the binding of the carboxylated CNTs with pTry. The MD simulation results demonstrated that the carboxylated CNTs adsorb to the enzyme stronger than the CNT without the–COOH groups. Our observations can provide an example of the nanoscale toxicity of COOH-f-MWCNTs for proteins, which is a critical issue for *in vivo* application of COOH-f-MWCNTs.

## Introduction

Carbon-based nanomaterials have unique atomic conformation, high volume-surface ratio, high electrical conductivity, good optical features, mechanical stability, and easy functionalization capacity. Nowadays, they have attracted considerable attention in nanobiotechnological and nanomedical applications [1–6]. Carbon nanotubes (CNTs) are widely used in different fields of nano-biosensors, biomolecular recognition devices, drug and gene delivery, and cancer therapy [7–11]. The excellent surface areas and layered and hollow structure of CNTs show that they can be ideal supporting drug molecules or gene transports via chemical or physical adsorption. Therefore, investigations of interactions of CNTs with drugs and biomolecules have increased biosafety issues for *in vivo* therapeutic applications [12–15]. However, the introduction of CNTs into biological systems can raise some questions about the potential risks of CNTs in futuristic medical and biological applications [16–20]. Despite astounding advances in industrial applications, the poor solubility of CNTs in water drastically limits effective biomedical applications. Hence, functionalization of CNTs makes them amenable for further binding of additional biomolecules, significantly increasing their biochemical and biophysical properties [21–23].

Previous studies have shown that adsorption of bio-macromolecules such as nucleic acids and proteins onto the surface of CNTs determines their cellular uptake and modulates the toxicity of CNTs [24–30]. The surface effects of CNTs on the structure and activity of proteins depend on their size, curvature, and chemistry [14, 31, 32]. To date, there are numerous studies on the interactions of CNTs with proteins [25, 27, 33, 34]. These studies have indicated that CNTs create disorders in the normal function of proteins and have some adding or reducing features, which are specific to proteins. Therefore, CNTs can affect the normal performance of proteins.

**Fig 1:**
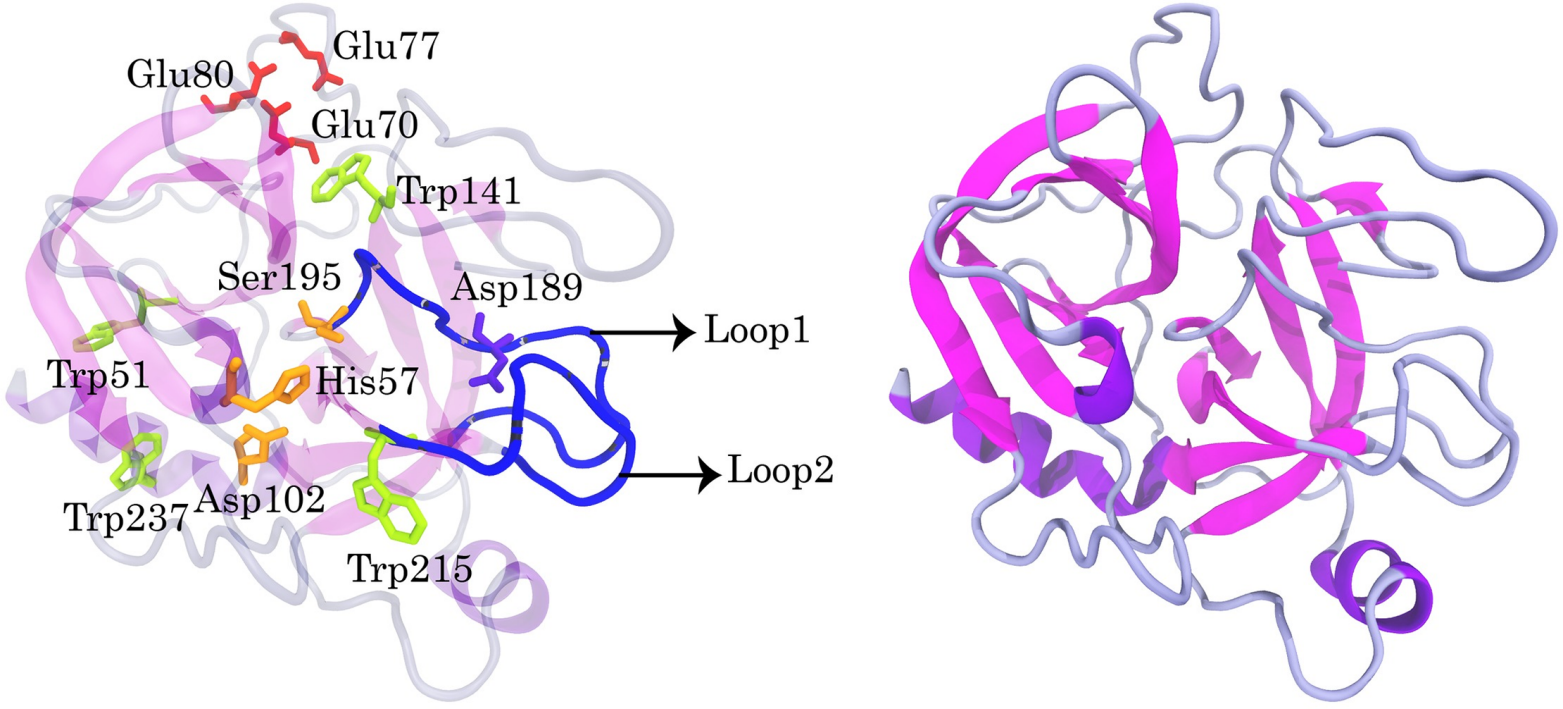
Left, the positions of the catalytic triad, tryptophan residues and structure of the S1 pocket of trypsin consists of residues 184–195 (Loop 1), 213–228 (Loop 2). Loop 3 (169–175) is not shown. Right, the native structure of trypsin (PDB ID: 1FNI).

The porcine trypsin (Enzyme Commission (EC) Number 3.4.21.4) is an important serine protease enzyme. It expresses in the pancreatic cells as an inactive proenzyme (called Trypsinogen) and has 223 amino acid residues arranged in two six-stranded beta barrels [35]. The residues His-57, Asp-102, and Ser-195 were located in the active site of pTry. According to previous studies, the residues 184–195 (Loop1), 213–228 (Loop2), and 169–175 (Loop 3) form the S1 pocket in the enzyme. The pocket contains a negatively charged residue (Asp-189), which stabilizes the side chains of the positively charged residues in the substrate (Fig 1). It was concluded that the pocket plays a vital role in the substrate selectivity [36].

The enzyme is extensively applied in different fields, including physiological processes, apoptosis, haemostasis, signal transduction, immune response, and food industry [37]. Previous studies have demonstrated that various nanoparticles can affect the enzymatic activity of pTry [38–40]. To the best of our knowledge, data has not been found about the potential impact of COOH-f-MWCNTs on the enzyme structure and activity. Considering the various uses of carbon nanotubes in medical and industrial applications, the study of the effect of COOH-f-MWCNTs on the trypsin enzyme seems to be necessary. In this work, we evaluate the mechanisms of interaction between COOH-f-MWCNTs and pTry by CD, fluorescence, UV–visible absorption spectroscopies, and MD simulations. Using a combination of experimental and theoretical techniques, we found that COOH-f-MWCNTs decrease enzyme activity in a dose-dependent manner and significantly influence the overall structure of the enzyme. The experimental and MD simulations results used in this study can provide a good perspective on the collision of trypsin with carboxylic-functionalized multi-walled carbon nanotubes.

## Materials and methods

### Materials

The porcine trypsin (Merck, Darmstadt, Germany) was dissolved in 20 mM Tris (2-Amino-2-(hydroxymethyl)-1, 3-propanediol, Trometamol, THAM, Tris base) buffer, pH 8.0 to form a 5.0 × 10^−5^ M^-1^ solution. The casein solution (1% (w/v)): 0/5 g of casein (Merck, Darmstadt, Germany) was suspended in 40 ml of Tris buffer and was entirely dissolved by placing on a water bath for 20 min. The solution was cooled and made up to 50 ml with Tris buffer. Trichloroacetic acid (TCA 10% (w/v)): 5 g TCA (Sigma, St. Louis, MO, USA) was dissolved in 50 ml dH_2_O. COOH-f-MWCNTs (OD: 20–30 nm, Length: 10–30 μm, SSA: >110 m^2^/g, Content of -COOH: 2.73 wt % were dissolved in ultrapure water (based on the TEM image of S1 Fig) using an urtra-sonicator prior to the experiment. Purity Multi-walled nanotubes>98wt %) were obtained from Iranian Nanomaterials Pioneers, Mashhad, Iran. Tris was purchased from Sigma (St. Louis, MO, USA).

### Molecular dynamics simulation

**Fig 2:**
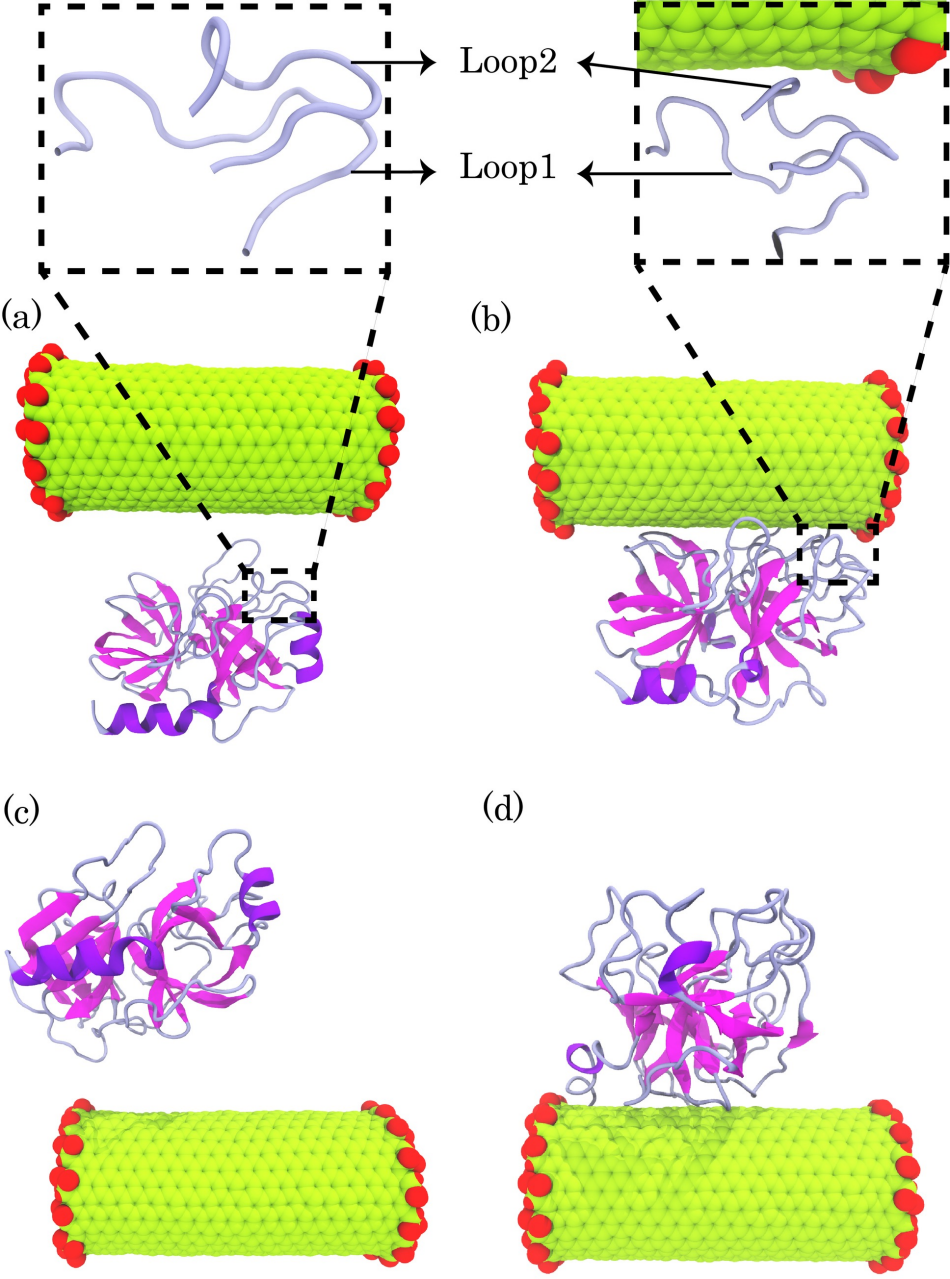
(a) and (b) the first and the last snapshots of trajectory in system 1, respectively. (c) and (d) the first and last snapshots of trajectory in system 2, respectively.

The initial coordinates of pTry were obtained from the Protein Data Bank (PDB ID: 1FNI) (Fig 1) [35]. A double-walled carbon nanotube (DWCNT) (the armchair with a (9, 9) inner tube and (14, 14) outer tube) with terminal carboxylation was used. The carbon atoms were modelled as uncharged Lennard-Jones particles, and described using parameters for the atoms of aromatic carbon. Four distinct simulation systems for COOH-f-DWCNT-pTry, each consisting of a COOH-f-DWCNT and a pTry molecule were prepared. In system 1, COOH-f-DWCNT was placed at an appropriate distance from the S1 pocket (Fig 2). In system 2, COOH-f-DWCNT was positioned in the opposite direction to system 1 (Fig 2). The direction of COOH-f-DWCNT in this system was in the same orientation as the docking calculation results. In system 3 and system 4, COOH-f-DWCNT was placed in the different relative orientations (S2 Fig). To identify the effects of the–COOH functional groups on the enzyme structure, four distinct simulation systems containing the non-functionalized DWCNT ((the armchair with a (9, 9) inner tube and (14, 14) outer tube)) and pTry were also prepared (S3 Fig). The orientation of DWCNT to the enzyme in the Model 1, Model 2, Model 3 and Model 4 was the same as the system 1, system 2, system 3 and system 4, respectively. To obtain comparative results, the enzyme was simulated in pure water. All MD simulations were performed using the GROMACS simulation software, version 5.1.2 [41, 42] with the OPLS/AA force field [43]. The enzyme and the enzyme-DWCNT complexes were solved in a box (with dimensions 10nm×10nm×10nm) using the extended simple point charge (SPC/E) [44] water model. The sufficient numbers of sodium (Na+) and chloride (Cl−) ions were used to neutralize all systems. The pressure and temperature were kept close to the intended values (1 bar and 300 K) using the Berendsen algorithm with τ_P_ = 0.5 and τ_T_ = 0.1 and ps, respectively [45]. All bonds lengths were maintained constant with the LINCS algorithm [46]. For all non-bonded interactions, a short-range spherical cut off 1 nm were used. The long-range interactions were calculated by the Particle Mesh Ewald (PME) method [47]. To energy minimization of the system, the steepest descent algorithm was applied. After minimizing the systems, the canonical (NVT) ensemble was conducted for 300 ps. All MD simulation systems were then equilibrated using an isothermal−isobaric (NPT) for 500 ps. The simulation length for each COOH-f-DWCNT-enzyme and DWCNT-enzyme system was 200 ns and 50 ns, respectively.

### Binding free energy calculations

The binding free energy (*ΔG*_*binding*_) between pTry and CNTs was calculated by the molecular mechanics/Poisson- Boltzmann Surface Area (PBSA) method [48]. The binding energy was obtained using the following equation:

$${\Delta G}_{b}={\Delta G}_{complex}-({\Delta G}_{pTry}+{\Delta G}_{CNT})$$ (1)

where Δ*G_b_* is the MM-PBSA binding energy, Δ*G_complex_* is the total free energy of the CNT-pTry complexes, and Δ*G_pTry_* and Δ*G_CNT_* are the total free energy of pTry and CNTs, respectively. The polar binding energy (Δ*G_polar binding_*), non-polar binding energy (Δ*G_non–polar binding_*), and total binding energy (**Δ*G***_*binding*_) were calculated by the following equations

$${\Delta G}_{polar\;binding}={\Delta G}_{electrostatic}+{\Delta G}_{pols}$$ (2)

$${\Delta G}_{non-polar\;binding}={\Delta G}_{van\;der\;Waals}+{\Delta G}_{npols}$$ (3)

$${\Delta G}_{binding}={\Delta G}_{polar\;binding}+{\Delta G}_{non-polar\;binding}$$ (4)

where Δ*G_pols_* is the electrostatic part of the free energy of solvation and Δ*G_npols_* is the non-electrostatic part of the free energy of solvation.

### Fluorescence measurements

All fluorescence spectra were recorded on a Cary eclipse fluorescence spectrophotometer (Carry eclipse, Varian, Australia) equipped with a 10-mm quartz cell and a 150-W xenon lamp. The photo multiplier tube (PMT) voltage was medium, and the excitation and emission slit widths were set to 5.0 nm. The excitation wavelength was adjusted to 280 nm and the emission wavelength scans ranged from 290 to 600 nm. The scan speed was 600 nm.min^−1^. Intrinsic fluorescence was recorded using 2.0×10^−5^ M^-1^ of pTry in 20 mM Tris, pH 8.0, in the absence and presence of different concentrations of COOH-f-MWCNTs. In fluorescence measurements, all spectra containing the COOH-f-MWCNTs-pTry solutions were subtracted with the COOH-f-MWCNTs solution spectra and were smoothed.

In this regard, the binding parameters determined with the Stern–Volmer plot by using Eq 5:

$$\frac{F_{o}-F}{F}=k_{q}\times \tau_{o}[Q]=1+K_{sv}[Q]$$ (5)

where *F_o_* and *F* define the steady-state fluorescence intensities in the absence and presence of a quencher, respectively. *k*_*q*_ is the quenching rate constant of the enzyme, and τ_0_ (10^−8^ s) is the average life time of the enzyme without any quencher, [Q] is the concentration of the quencher, *K*_*SV*_ is the Stern–Volmer quenching constant, [49] and other form of the Stern-Volmer equation is 6: [50]

$$Log\;\left[\frac{F_{o}-F}{F}\right]=LogK_{A}+n\;Log[Q]$$ (6)

and thermodynamic Eq 7:

$${\Delta G}^{0}=-RT\;K_{A}$$ (7)

K_*A*_ is the binding constant, and *n* is binding sites of the COOH-f-MWCNTs-pTry complex, ΔG^0^ is the free energy change, *R* is the gas constant (8.314 Jmol^-1^k^-1^), and *T* is the absolute temperature.

### UV–visible absorption measurements

All UV-visible absorption spectra were recorded on a Nanodrop 2000 spectrophotometer (Thermo Scientific™ Nanodrop™) equipped with a 10-mm quartz cell. The UV–visible spectra of pTry in the absence and presence of COOH-f-MWCNTs were recorded in the range of 190–420 nm at 298 K. The enzyme concentration was 2.0 × 10^−5^ M^-1^ for the experiment. In the UV–visible absorption measurements, all spectra containing the COOH-f-MWCNTs-pTry solutions were subtracted with the COOH-f-MWCNTs solution spectra and were smoothed.

### Far-UV circular dichroism measurements

The CD spectra were operated over the range of wavelength of 190–250 nm with a J-810 CD spectropolarimeter (Jasco, Tokyo, Japan) using a quartz cell with a path length of 1 mm. The scanning speed was set to 200 nm.min^−1^. The results were reported as molar ellipticity [θ] (degree cm^2^.dmol^-1^), based on the average amino acid residue weight (MRW) of pTry and the data were smoothed using the Jasco J-810 software, which includes a fast Fourier-transform noise reduction routine. The secondary structure contents of the enzyme in percentage were estimated by using the software provided by the JASCO software version 1.10.02. In CD spectroscopy studies, all spectra containing the COOH-f-MWCNTs-pTry solutions were subtracted with the COOH-f-MWCNTs solution spectra and were smoothed.

### Protease activity assay

The activity of pTry was measured at pH 8.0 in 20 mM Tris buffer at room temperature. The enzymatic reaction was evaluated with 100 μg.ml^-1^ pTry concentration and 1% casein as substrate in the final volume of 500 μl. After 10 minutes incubation, the enzymatic reaction was stopped by addition of 500 μl of 10% TCA and then the supernatant absorbance was determined in 280 nm [51]. The activity of protease can be determined as the quantity micromoles of tyrosine released from substrate by the enzyme in per minute under the assigned assay conditions [52].

### Enzyme kinetics

The Michaelis–Menten kinetics of native pTry and apparent kinetics of the enzyme in the present of COOH-f-MWCNTs were determined. A solution of casein in 20 mM Tris buffer (pH 8.0) was serially diluted to establish a substrate range from 0 to 15 mg.ml^-1^. The enzyme was added to individual tubes containing the casein solutions in the absence or presence of COOH-f-MWCNTs. The reaction was allowed to proceed for 10 minutes at room temperature. The absorbance was measured at 280 nm and the acceleration of product alteration was designated with the use of the primitive rate of the reactions. By using the Michaelis–Menten equation (GraphPad, prism 6, USA) and plotting reaction rate (V) against concentration of the substrate, the parameters (*K*_m_ and *V*_max_) were obtained. The *k*_cat_ was calculated by using the following equation:

Vmax/[E] (8)

where [*E*] define as the initial enzyme concentration. (The molar mass of pTry was considered 23,300 g.mol^-1^).

### Unalterable thermo-inactivation of pTry

The inconvertible heat inactivation of the enzyme was accomplished by placing of the pTry samples at the temperature 313 K in the absence and presence of COOH-f-MWCNTs. For thermal stability evaluation, variant concentrations of COOH-f-MWCNTs were examined on the thermostability of the enzyme. At regular intervals, the samples were removed and cooled on ice for 30 minutes and the residual activity was then determined at 298 K under the assay conditions. In each experiment, the residual activity of the enzyme that incubated at 40°C for 0 min considered as control (with 100% activity). The *k*_*in*_ and the half-life the enzyme inactivation were determined as follows:

$$\mathrm{Ln}\left(Activity\right)=\mathrm{Ln}(Activity{)}_{0}-K_{in}t$$ (9)

and

$$Half-life=0.693/K_{in}$$ (10)

### Statistical analysis

All experiments were performed in triplicate and data are shown as mean ± SD. Statistical analysis was performed according to the Student’s t -test and one-way ANOVA analysis followed by the Dunnett’s multiple comparison tests. The probability values of P <0.05 were considered as significance.

## Results and discussion

**Fig 3:**
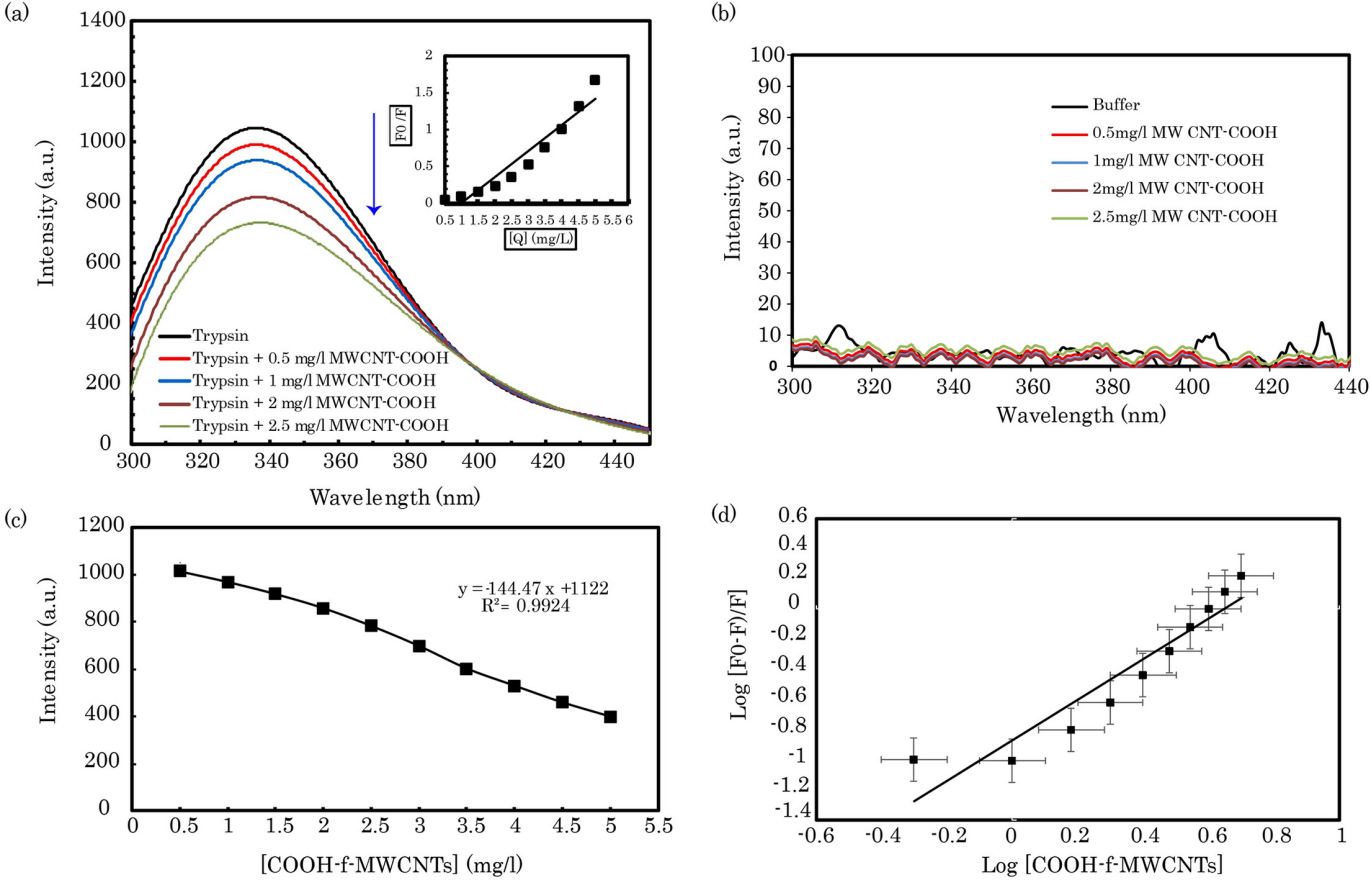
(a) Fluorescence spectra of the COOH-f-MWCNTs-pTry system. Conditions: λ_ex_ = 280 nm, pH 8.0; *C*_trypsin_ 2.0 × 10^−5^ M, *C*_CNTs_ are in the total volume of the reaction mixture; pH 8.0, T = 298 K. (b) Emission spectra of COOH-f-MWCNTs systems alone. (c) The plot of fluorescence quenching for trypsin with the concentration of COOH-f-MWCNTs. (d) The logarithmic plot of trypsin with the concentration of COOH-f-MWCNTs. Data are shown as mean values ± S.D.

First, steady state fluorescence was performed to obtain data on the tertiary structural properties of pTry. The porcine trypsin is a globular protein that contains four tryptophan (TRP) residues (51, 141, 215, and 237), which are used as intrinsic fluorophores (Fig 1) [53]. To examine the conformational properties of pTry in the aqueous COOH-f-MWCNT solution, the enzyme molecules were allowed to interact with various concentrations of COOH-f-MWCNT, and the emergent fluorescence emission maxima were measured (Fig 3). In our investigations, the fluorescence emission of pTry was obtained at 339 nm, upon excitation at 280 nm. Available data indicate that the fluorescence intensity of the enzyme decreases upon addition of various concentrations of COOH-f-MWCNTs, while the fluorescence emission spectra of the enzyme exhibit a slight blue shift upon addition of COOH-f-MWCNTs (Fig 3(A)). COOH-f-MWCNTs had not significant emission in fluorescence measurements (Fig 3(B)). It is clear that the fluorescence of pTry was obviously quenched after the addition of COOH-f-MWCNTs (Fig 3(C)). To obtain this quenching mechanism, the results were computed with the Stern–Volmer equation (inset upper right in Fig 3(A)). The plot, at various concentrations of COOH-f-MWCNTs was linear, which suggests that the type of quenching of pTry follows single mechanism kinetics. As shown in Fig 3(C), the *k*_*SV*_ and *k*_*q*_ (*k*_*q =*_ *k*_*SV*_/τ_0_) values of the COOH-f-MWCNTs-pTry complex were obtained as 0.352 (×10^8^ M^-1^) and 0.352 (×10^16^ M^-1^.s^-1^), respectively. In this study, the order of magnitude of *k*_*q*_ was 0.352×10^16^. However, the maximum scatter collision quenching constant, *k*_*q*_, of various quenchers with the biopolymer was 2×10^10^ (M^-1^.s^-1^) [54]. Therefore, static quenching drove the fluorescence quenching process, which is associated with the formation of the ground state complex between the enzyme and COOH-f-MWCNTs, in compatibility with the UV-visible absorptions. Eq 9 was used to obtain the binding parameters. Fig 3(D) shows the plots of log [(*F*_0_-*F*)/*F*] versus log [COOH-f-MWCNTs]. The values of slope and intercept were 1.3 and –0.871, respectively. Since the molecular weight of COOH-f-MWCNTs was not definite in the COOH-f-MWCNTs-pTry samples, we used λ as a pseudo molar concentration pursuant to previous studies [50]. According to the size of COOH-f-MWCNTs in this work (OD: 20–30 nm; length: 10–30 μm), the value of λ was no more than 1×10^−8^. The value of intercept was -0.871, which revealed that *K*_*A*_ is equal to 0.135/λ. The numerical value of *n* indicates that there is a significant binding site for COOH-f-MWCNTs on pTry. On the other hand, the value of *K*_*A*_ (1.35 ×10^7^ M^-1^) shows that there are strong interactions between COOH-f-MWCNTs and enzyme: these are significantly stronger than the interactions between HO-MWCNTs and bovine serum albumin (BSA) (1.39 ×10^4^ M^-1^) in the same studies [55]. The value of *ΔG*^*0*^ in the formation of the COOH-f-MWCNTs-pTry complex was –40 kJ.mol^-1^. This value indicates a spontaneous exothermic reaction, as expected. The fluorescence quenching of the enzyme demonstrates that the tryptophan residues in the hydrophobic core were gradually exposed to the aqueous medium with increase in the concentration of COOH-f-MWCNTs. In addition, the small blue shift in the position of the fluorescence emission spectra indicates that the COOH-f-MWCNTs-pTry interactions might occur around the tryptophan residues, which were found to be located in a more hydrophobic microenvironment after the addition of COOH-f-MWCNTs [56–58].

**Fig 4:**
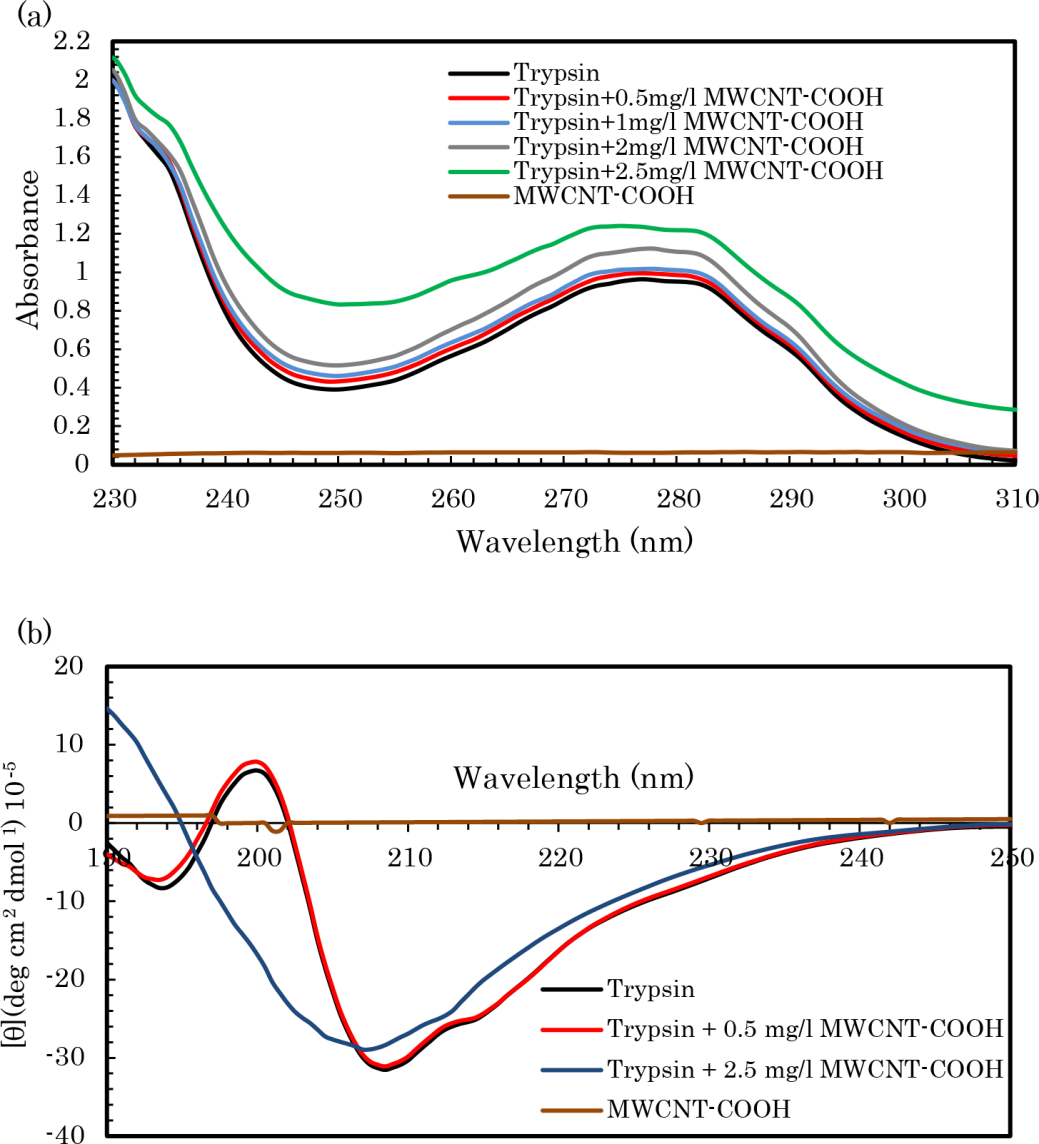
(a) UV–visible absorption spectra of trypsin. *C*_trypsin_ = 2.0 × 10^−5^ M, *C*_CNTs_ are in the total volume of the reaction mixture; pH 8.0, T = 298 K. (b) CD spectra of trypsin and COOH-f-MWCNTs-pTry mixtures. *C*_trypsin_ = 1 × 10^−4^ M. *C*_CNTs_ are in the total volume of the reaction mixture; pH 8.0, T = 298 K. Data are shown as mean values ± S.D.

To study the effects of COOH-f-MWCNTs on the pTry structure, UV-visible absorption was used. The aromatic amino acid residues of pTry contribute to UV absorbance at approximately 274 nm with respect to their π-π* transition [59]. Therefore, the UV absorption peak around 278 nm suggests the buried aromatic amino acid residues of the enzyme (Fig 4(A)). The UV-visible spectra indicate that increase in the concentration of COOH-f-MWCNTs in the pTry solution significantly increase the intensity of the UV absorption spectra. It suggests that COOH-f-MWCNTs and pTry form a complex, as the pTry molecules get adsorbed on the surface of COOH-f-MWCNTs: this indicates that the side chains of residues were partly changed [3, 60]. These results confirm that the static quenching mechanism is in high agreement with the fluorescence findings.

CD is an important tool for rapid monitoring of the folding and unfolding states and secondary structures of proteins in solutions [61–63]. The far-UV CD spectra of proteins (185−250 nm wavelengths) were used to provide data about the composition of the secondary structure components [63]. To obtain further information about the effects of COOH-f-MWCNTs on the secondary structural contents of pTry, the CD spectra (far-UV) were used for two COOH-f-MWCNTs-pTry mixtures (0.5 and 2.5 mg. L^-1^). From CD spectra analysis, it is clear that the alpha-helix structures decreased upon increase in the COOH-f-MWCNTs concentration (Fig 4(B)). Table 1 indicates that COOH-f-MWCNTs could induce the secondary structure changes in pTry by the distribution of hydrogen bonding networks.

**Fig 5:**
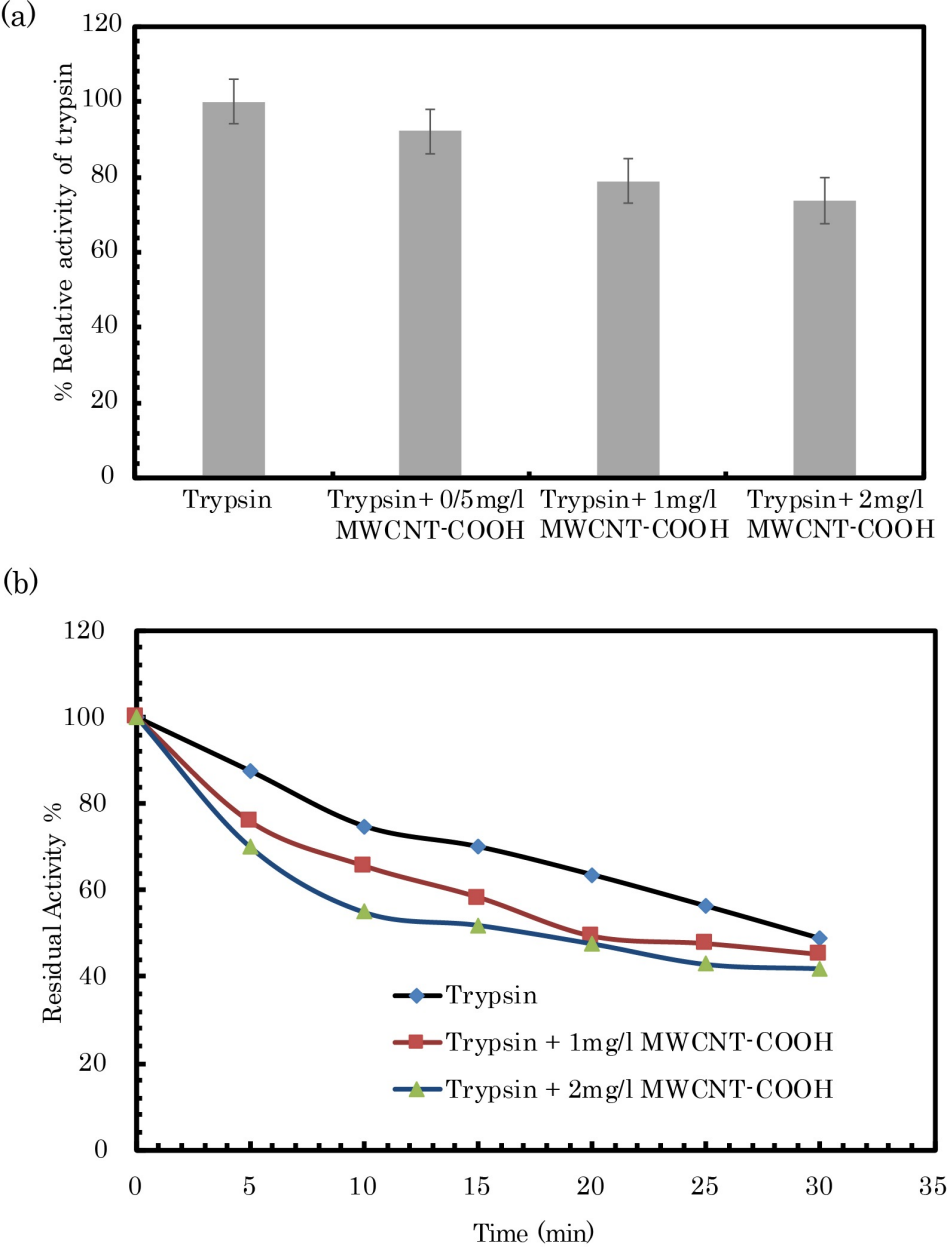
(a) Effects of COOH-f-MWCNTs on the activity of trypsin. (b) The thermostability of trypsin in the presence of COOH-f-MWCNTs. pH 8.0, T = 330 K: *C*_trypsin_ (control) = 100 μg/ml. *C*_CNTs_ are in the total volume of the reaction mixture; pH 8.0, T = 298 K. Data are shown as mean values ± S.D.

The relative activity of the enzyme was determined in the presence of different concentrations of COOH-f-MWCNTs (0–2 mg. L^-1^) (Fig 5(A)). As can be observed, the relative activity of pTry was approximately 92%, 79%, and 74%, in the presence of 0.5 mg/l, 1 mg/l, and 2 mg/l of COOH-f-MWCNTs, respectively.

**Table 1:** The influence of COOH-f-MWCNTs on the secondary structure of trypsin.

System (experimental)	α-Helix (%)	β-Sheet (%)	Random coil (%)	Others
**Trypsin**	16.2	34.7	28.0	21.1
**Trypsin+0.5 mg.L^-1^ CNT**	16.0	35.6	28.9	19.5
**Trypsin+2.5 mg.L^-1^CNT**	5.2	59.9	34.9	-

The enzyme showed a Michaelis-Menten type of kinetics when hydrolyzed with casein in the absence and presence of COOH-f-MWCNTs. As shown in Table 2, the kinetic parameters do not undergo significant variations after addition of COOH-f-MWCNTs: only a slight increase and decrease in *K*_*m*_ and *k*_*cat*_ of pTry is observable. This results in slight decrease in *k*_*cat*_*/K*_*m*_, in the COOH-f-MWCNT aqueous solution as compared to the free form. The results of the enzyme kinetics show that increase in the *K*_*m*_ value may be due to diffusion limitations and steric hindrance of the active site. Decrease in *k*_*cat*_ could be due to the restricted diffusion of the substrate to the active site and/or the higher structural rigidity of the enzyme due to the presence of the nanotube [64].

**Table 2:** Effect of COOH-f-MWCNTs on the kinetic parameters of the porcine trypsin. The values were calculated using GraphPad Prism.

System	K_m_ (mg.ml^-1^)	*k*_*cat*_ (min^-1^)	*k*_*cat*_/K_m_)	*R square*
**Trypsin**	3.9	37.79	9.7	0.988
**Trypsin+2.5 mg.L^-1^ CNT**	4.24	33.72	8.0	0.993

The effect of COOH-f-MWCNTs on the thermal stability of pTry has also been studied. In the presence of two concentrations of COOH-f-MWCNTs, the pTry enzyme exhibits decrease in thermostability at 330 K compared with the free form (Fig 5(B)). The plots of the Ln of residual activity versus time are linear. Therefore, a first-order decay process under these conditions was observed. The half-life of the enzyme was 9.65 min in the free form, while in the presence of 1 and 2 mg. L^-1^ of COOH-f-MWCNTs it was 8.68 min and 7.98 min, respectively (Table 3).

**Table 3:** Inactivation rate constant (*k*_*in*_) and half-life of the trypsin in the absence and presence of COOH-f-MWCNTs at T = 330 K.

System	*k*_*in*_ *(min*^*-1*^*)*	*Half-life (min)*	*R square*
**Trypsin**	0.0718	9.65	0.973
**Trypsin+1 mg.L^-1^ CNT**	0.0798	8.68	0.979
**Trypsin+2mg.L^-1^ CNT**	0.0868	7.98	0.954

**Fig 6:**
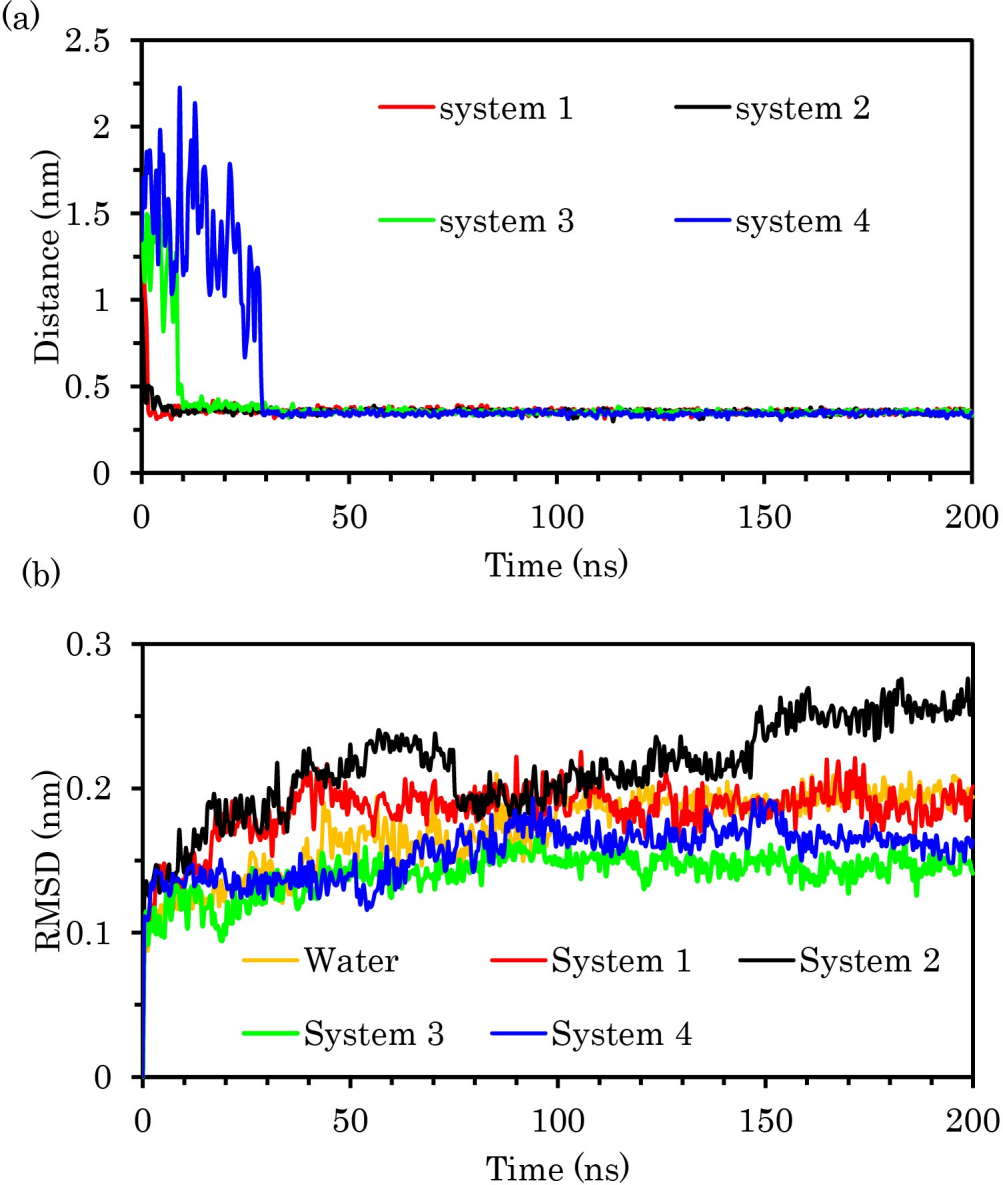
(a) Center of mass (COM) distances between the enzyme and COOH-f-DWCNT as a function of time (b) The RMSD of C-α of trypsin during the simulation time.

To provide information about the enzyme adsorption on the COOH-f-DWCNT surface, we calculated the center of mass (COM) distance between the enzyme and COOH-f-DWCNT during MD simulations (Fig 6(A)). As demonstrated, during the early steps of the simulations the distances between COOH-f-DWCNT and the enzyme decreased drastically in all the systems, indicating the early adsorption of the enzyme on the surface of COOH-f-DWCNT. We also computed the C_alpha_ root-mean-square-deviation (RMSD) of the enzyme for determining the stability of the enzyme over the simulations (Fig 6(B)). As can be seen in system 2, the RMSD values of pTry increased in the presence of COOH-f-DWCNT, which indicates rapid divergence from the starting structure. This observation demonstrated that the fluctuations of pTry were more in system 2 than in other systems (Fig 6(B)). In system 1, there was no significant difference between the RMSD values of the system and the enzyme in water. However, the RMSD values for the enzyme in systems 3 and 4 were approximately the same as each other and the enzyme was more stable than the other systems during the simulation time. Based on the RMSD results, it can be suggested that the results of the MD simulations depend on the starting position of COOH-f-DWCNT.

**Fig 7:**
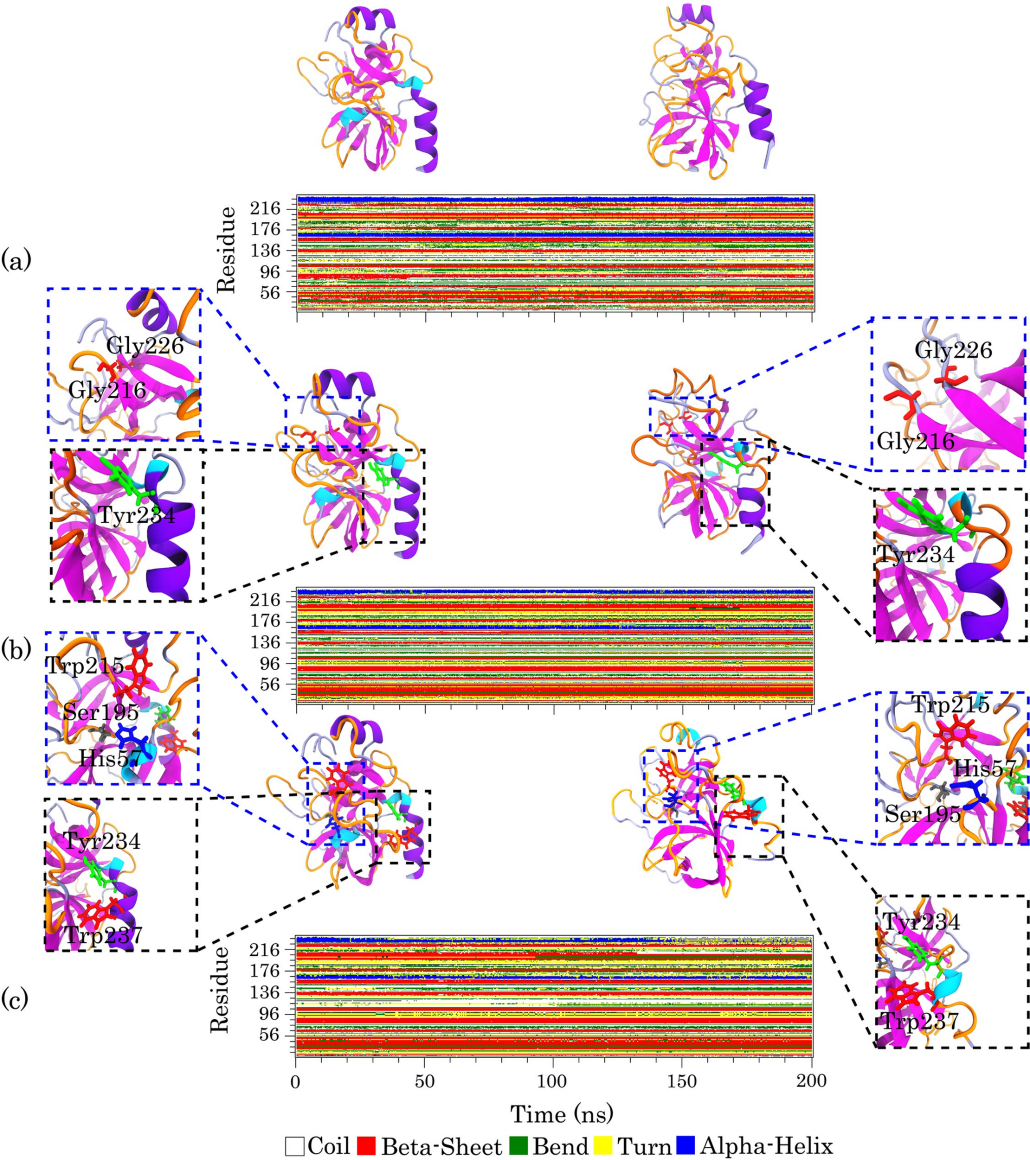
Time evolution of the secondary structure of the enzyme during MD simulations generated by DSSP for (a) the enzyme in water (as control), (b) system 1, and (c) system 2. The cartoon model of the first and the last snapshots of MD of the enzyme structure are shown in left and right respectively. Changes of secondary structure of several residues are shown, for instance. The alpha helix, 3_10_ helix, beta sheet, turns, and coil structures are colored in violet, cyan, magenta, orange, and ice blue, respectively. For the DSSP analysis of the enzyme in system 3 and system 4, refer to the supplementary information.

**Fig 8:**
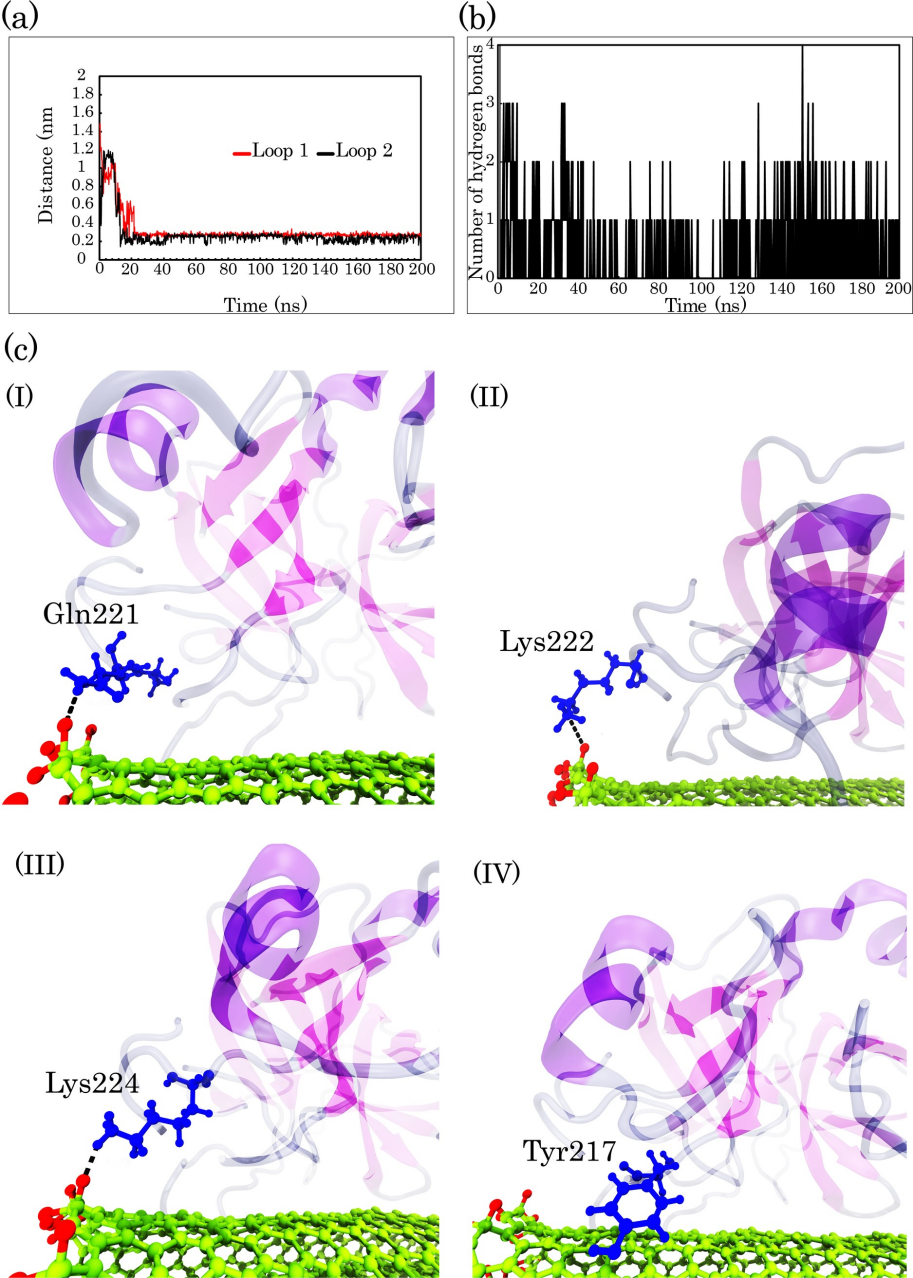
MD simulations indicate interaction between COOH-f-DWCNT and S1 pocket of the enzyme. (a) Minimum distance between Loop 1 and Loop 2 of S1 pocket with COOH-f-DWCNT in system 1 over the simulation time (for analysis of minimum distance between Loops with COOH-f-DWCNT in system 3, refer to the supplementary information). (b) The number of hydrogen bonds between S1 pocket and COOH groups of COOH-f-DWCNT in system 1 as function of time. (c) Local snapshots from hydrogen binding interaction by (I) Gln-221, (II) Lys-222, and (III) Lys-224 and π-π stacking interaction by (IV) Tyr-217.

By calculating Define Secondary Structure of Proteins (DSSP) [65] from the MD simulation, we found that there was a notable decrease in the alpha-helix contents of pTry upon interaction with COOH-f-DWCNT. This was in agreement with our experimental results (Fig 7) and with previous studies [66, 67]. To further validate the DSSP findings, we also obtained the residue secondary structure of the enzyme in each system using the STRIDE web server [68]. Zhao et al. reported that the carboxylated CNTs increase the beta-sheet structures and decrease the alpha-helical contents of alpha-chymotrypsin [69]. According to the data obtained from DSSP and the STRIDE server, over the MD simulation time, the α-helices of the enzyme were unstable and adopted the 3_10_-helix, coil, and turn structures (Fig 7 and S4 Fig). Because of the docking method could anticipate the preferred direction of the carbon nanotube to the enzyme and since the importance of the S1 pocket of the enzyme, we focused on systems 1 and 2. The MD simulation analyses for the other orientations and systems were provided in the supplementary information. As demonstrated in Fig 7, Tyr-234 and Trp-237, which are located in the alpha-helix structure of the enzyme, lost the helicity and the beta-sheet structure containing Trp-215 was changed to turn. Therefore, the quenching and slight blue shift observed for pTry—discussed earlier- could be due to the interactions, which increase the local concentrations of COOH-f-MWCNTs around the tyrosine and tryptophan residues of pTry. Meanwhile, in all four COOH-f-DWCNT systems, pTry lost its 3_10_-helix structure consisting of residues 56 to 58 (“AHC”), where His-57 in the active site of the enzyme adopted a turn structure. The specificity of pTry is usually determined by the residues Asp-189 (Loop 1), Gly-216, and Gly-226 (Loop2) [36]. As indicated in Fig 7, Gly-216 and Gly-226 (in system 1) with structure of turn and beta-sheet respectively adopted the coil structure. These data suggest that COOH-f-DWCNT induced a significant change in the overall conformation of pTry. In systems 1 and 3, although COOH-f-DWCNT had the different starting orientation to the enzyme, the S1 pocket was blocked by COOH-f-DWCNT (Fig 2 and S2 Fig). In general, COOH-f-DWCNT, independent from its initial orientation, was adsorbed to the two directions of the enzyme (the docking results orientation and the opposite direction), indicating that the mentioned two directions of the enzyme are the adsorption sites for COOH-f-DWCNT. In system 1, Loop 3 did not show significant interactions with COOH-f-DWCNT: therefore, we focused on the structural changes in the Loop 1 and Loop 2 of the S1 pocket, whereas in system 3, Loop 2 and Loop 3 remarkably interact with COOH-f-DWCNT (S2 Fig). We first calculated distance between the center of mass (COM) of COOH-f-DWCNT and the Loops in system 1 (Fig 8(A)). As can be seen in Fig 8(A), the distance of Loop 1 and Loop 2 from COOH-f-DWCNT decreases rapidly after 10 ns: compared to Loop1, Loop 2 is closer to the surface of COOH-f-DWCNT because the cationic residues in Loop 2 could interact with the COOH groups of COOH-f-DWCNT. We also calculated the number of hydrogen bonds between the S1 pocket and COOH-f-DWCNT in system 1 and system 3 over the simulation time (Fig 8(B) and S5 Fig). The results in Fig 8(B) show that the negatively charged carboxyl groups of COOH-f-DWCNT can form hydrogen bonds with the polar residues of the S1 pocket, such as the Gln-221, Lys-222, and Lys-224 residues.

The contribution energy per residue of the S1 pocket to the total binding free energies is listed in Table 4. These results confirm the role of residues of the S1 pocket in the absorption process. Eight residues of the S1 pocket were in contact with COOH-f-DWCNT, while most of them were basic and polar residues. The Lys-188, Lys-222, and Lys-224 residues with *ΔG*_*binding*_ < –90 kJ.mol^-1^ and Tyr-217 and Gln-221 with *ΔG*_*binding*_ < –8.0 kJ.mol^-1^ had more effective contributions to the total binding energy. In addition, the contribution energy of each S1 pocket residue in system 3 has been shown in the supplementary information (S1 Table).

**Table 4:** The binding energy (ΔG_binding_) of S1 pocket residues (kJ.mol^-1^) in system1.

Residue	ΔG_binding_	Residue	ΔG_binding_	Residue	ΔG_binding_
**Lys-188**	-92.3899	**Gly-219**	-3.8869	**Lys-222**	-110.388
**Gln-192**	-6.4745	**Cys-220**	-5.3121	**Lys-224**	-110.823
**Tyr-217**	-8.4082	**Gln-221**	-8.8456	-	-

The aromatic residue Tyr-217, which is located at Loop 2 of the S1 pocket, can interact with the aromatic rings on the surface of COOH-f-DWCNT by π-π stacking interactions (Fig 8(C)). We were able to demonstrate the representative snapshots of π-π stacking attractions and hydrogen bonds, which contributed to the interaction process between COOH-f-DWCNT and the S1 pocket (Fig 8(C)). These interactions can change the structure of the S1 pocket in consistence with the DSSP findings, and correspondingly influence performance of the active site. Similarly, a recent study has also shown that the interactions between the COOH groups of COOH-SWCNT and several specific basic and polar residues of the cytochrome P450 enzyme (CYP3A4) can inhibit its catalytic activity. The authors believe this suggests electrostatic interactions have an important role during the binding process [70].

Consequently, the results highlight that the S1 pocket adsorbs on the COOH-f-DWCNT surface through electrostatic interactions, and hydrogen bonds and Loop 2 plays a crucial role in the adsorption of the enzyme on the nanotube surface. As a result, a combination of different interaction types contributes to the blocking of the S1 pocket by COOH-f-DWCNT. Fig 2 shows representative snapshots of the first (a and c) and the last (b and d) steps of the trajectory in system 1. The figure supports the important role of Loop 2 in the adsorption process of the S1 pocket on the carbon nanotube. It is clear that conformational changes in the S1 pocket can influence substrate accessibility correspondingly. Overall, our results confirm that COOH-f-DWCNT adsorbs onto the S1 pocket with a strong tendency: it can be the reason for decrease in the activity of pTry in the presence of COOH-f-DWCNT (See S2 Fig for system 3).

**Fig 9:**
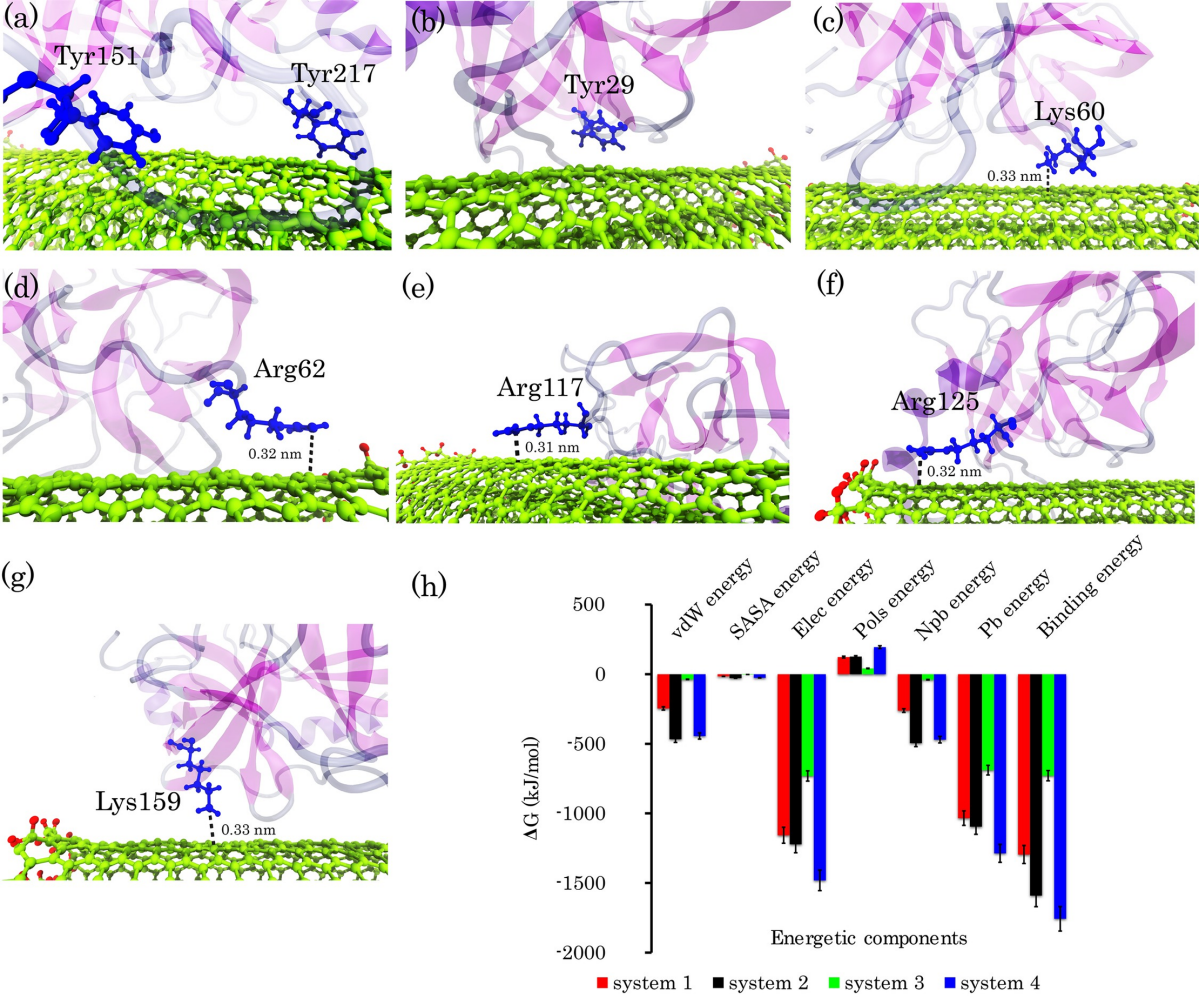
The snapshot of MD simulation outcomes shows the preferred binding sites on enzyme for COOH-f-DWCNT. π-π stacking interactions (a) Tyr 151 and Tyr 217 (system 1) (b) Tyr 29 (system 2). π–cation stacking interactions (c, d) Lys 60 and Arg 62 (system 1) (e, f, g) Arg 117, Arg 125 and Lys 159 (sysytem2). (h) Energetic components of COOH-f-DWCNT-pTry complex. vdW is van der Waals energy, SASA is non-polar energy, Elec is electrostatic energy, Pols is polar solvation energy, Npb is non-polar binding energy and Pb is polar binding energy.

Previous studies have shown that the π-cation interactions—the interactions between aromatic rings on the surface of carbon nanotube and the cationic group of basic residues—and π-π-stacking interactions—a non-covalent attractive force between two aromatic rings—contribute to the binding of CNT to proteins [70, 71]. As demonstrated in Fig 9, the guanidinium group of Arg-117, Arg-125 (in system 2), and Arg-62 (in system 1) and the amine group of Lys-60 (in system 1) and Lys-159 (in system 2) can participate in the multiple interactions—such as π-cation interactions and hydrogen bonds—with COOH-f-DWCNT (Fig 9). Additionally, the aromatic residues Tyr-151 and Tyr-217 (system 1) and Tyr-29 (system 2) can interact with the aromatic rings of COOH-f-DWCNT by the π-π-stacking interactions (Fig 9 panels (a) and (b)).

We computed the total binding energy, van der Waals, electrostatic and non-polar binding energies between COOH-f-DWCNT and the enzyme for all the MD simulations systems (Fig 9(H)). The results indicate that COOH-f-DWCNT strongly binds to the enzyme and that electrostatic interactions play important roles in interactions of the nanotube with the enzyme: this is in good agreement with previous studies [72–75]. The results also indicate that *ΔG*_*polar binding*_ (–1034.07 kJ.mol-1) in system 1, *ΔG*_*polar binding*_ (–1095.29 kJ.mol-1) in system 2, *ΔG*_*polar binding*_ (-689.68 kJ.mol^-1^) in system 3, and *ΔG*_*polar binding*_ (-1287.11 kJ.mol^-1^) in system 4 are favorable for the formation of the COOH-f-DWCNT-pTry complexes (Fig 9(H)), which are related to the COOH groups on COOH-f-DWCNT [76]. It may be worthwhile to point out that a combination of different interaction types contributes to the interaction process between COOH-f-DWCNT and pTry.

**Fig 10:**
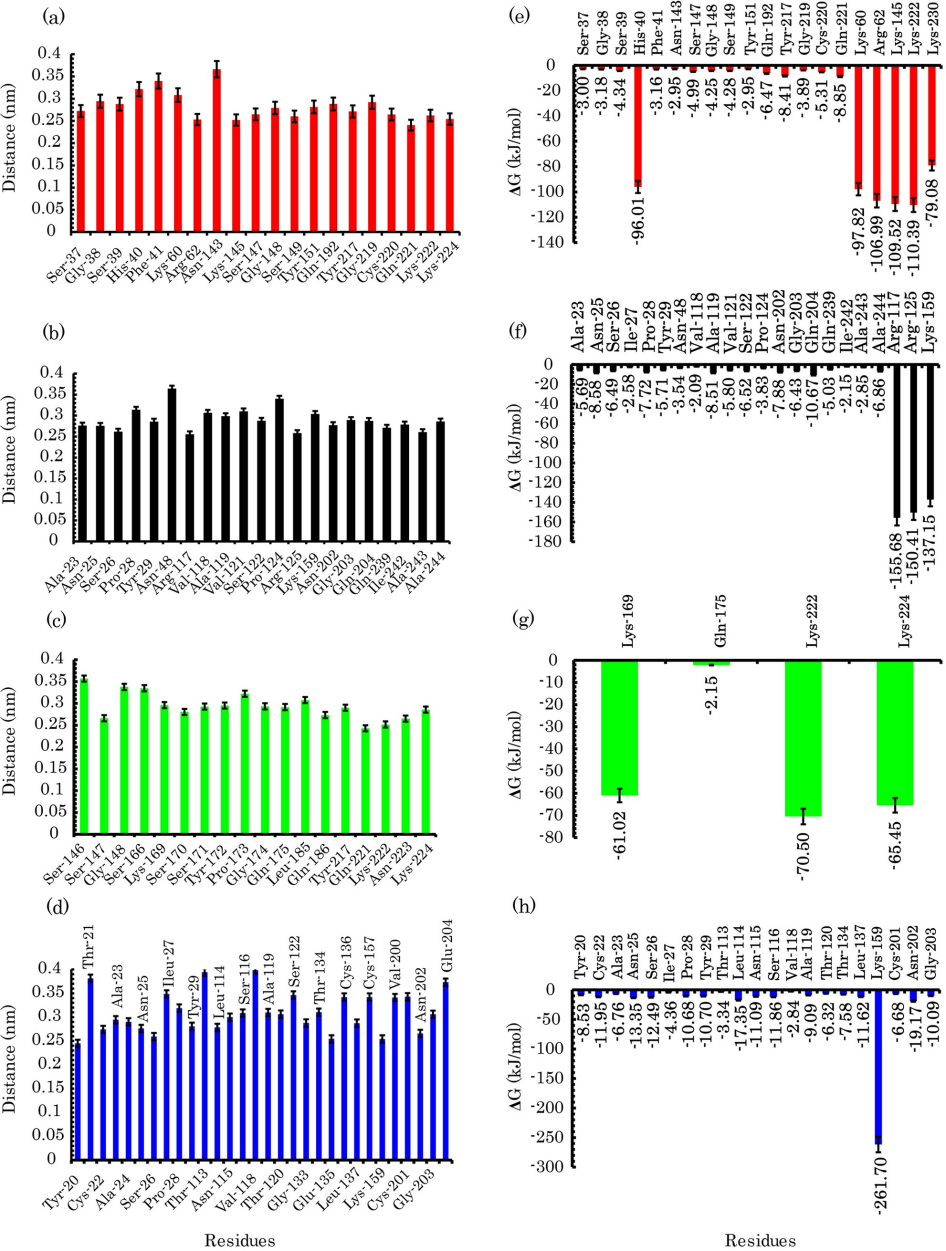
Adsorption process of the enzyme on the surface of COOH-f-DWCNT. (a) to (d) The minimum distance between residues and COOH-f-DWCNT in system 1, system 2, system 3 and system 4, respectively. (e) to (h) the energy residues with value of binding energy < -2 kJ.mol^−1^ in system 1, system 2, system 3 and system 4, respectively.

To gain more insight into the adsorption of pTry on the nanotube surface, we calculated the number of contacts between pTry and COOH-f-DWCNT (S5 Fig). As indicated, the number of contacts increased quickly in all the systems: this increasing trend in system 2 is more apparent during 200 ns simulation time. When the distance between one of the non-hydrogen atom residues and the CNT surface is less than 0.5 nm, the residues are identified as “contacted” and they can act as binding sites [71]. Therefore, we also computed the minimum distance and the total binding energy between each residue of the enzyme and the nanotube (Fig 10). It can be observed that a large number of residues have a distance < 0.5 nm with favorable *ΔG*_*binding*_ < -2 kJ.mol^-1^.

Recent MD simulations have shown that the guanidinium group of the arginine residues can intercede in protein-CNT interactions [72, 73, 77]. Eugen et al. stated that Arg-112 is the most important residue in the binding of lysozyme to the surface of CNT [72]. It has been shown that the interaction sites between human myeloperoxidase and COOH-CNT contain the arginine and tyrosine residues [78]. In excellent agreement with these studies, the minimum distance analysis and the energy calculations revealed that the positively charged residues, with a minimum distance of < 0.5 nm, could make major contributors to the binding free energy (Fig 10 and S2 Table). Our results also demonstrated that the polar residues of the enzyme, such as Ser-37, Ser-39, Asn-143, Ser-147, Ser-149, Gln-192, and Gln-221 in system 1 (Fig 10 (e)) and Asn-25, Ser-26, Asn-48, Ser-122, Asn-202, Gln-204, and Gln-239 in system 2 (Fig 10(F)), can interact with the carboxyl groups of COOH-f-DWCNT. The polar residue for system 3 was Gln-175 and the related polar residues for system 4 were Asn-25, Ser-26, Thr-113, Asn-115, Ser-116, Thr-120, Thr-134, and Asn-202. Collectively, the results in Fig 10 comply with previous studies and show that these aforementioned interactions play important roles in the COOH-f-DWCNT-pTry interactions.

To identify the effects of the carboxyl groups of CNT on the enzyme structure, we also modelled four systems containing the CNT without the–COOH functional groups and the enzyme. The initial CNT orientations to the enzyme were precisely the same as they were in the COOH-f-DWCNT-pTry systems (S3 Fig). We first compared the van der Waals interactions, the electrostatic interactions and the polar and nonpolar binding energies between the carbon nanotube and the enzyme in both COOH-f-DWCNT-pTry and DWCNT-pTry systems (S3 Table). The results indicate that electrostatic interactions and polar binding energies for the COOH-f-DWCNT-pTry systems were favourable, whereas the aforementioned interactions for the systems with CNT without the–COOH groups were equal to zero. Therefore, it can be suggested that the carboxyl groups of CNT could interact with the enzyme using the electrostatic and polar interactions. In other words, COOH-f-DWCNT had more interactions with the enzyme than DWCNT. Therefore, it can be concluded that COOH-f-DWCNT could affect the enzyme structure more than the CNT without the–COOH groups. Additionally, COOH-f-DWCNT had the ability to make hydrogen bonds with the enzyme, while the CNTs without the functional groups had not this capability (Fig 8(B) and S5 Fig). In summary, the results of the hydrogen bonds and the energies indicate that COOH-f-DWCNT adsorbs to the enzyme stronger than DWCNT.

## Conclusions

The interactions of COOH-f-MWCNTs with pTry were investigated in this paper by using experimental and MD simulation techniques. The results indicate that the inherent fluorescence intensity of pTry decreases in the presence of increased COOH-f-MWCNTs concentrations based on the static quenching mechanism. This is related to the formation of a COOH-f-MWCNTs-pTry complex. The CD results show that the alpha-helix structure content decreases in the presence of COOH-f-MWCNTs. Therefore, COOH-f-MWCNTs interact with the main chain of pTry and disturb the enzyme hydrogen bond networks. To ensure our experimental results, MD simulations and binding free energy calculations were done. The simulation analyses demonstrate that COOH-f-DWCNT binds to the S1 pocket of pTry and can block the active site of the enzyme. The MD simulations reveal that COOH-f-DWCNT increases the flexibility of pTry and the enzyme conformation. The binding free energy calculations demonstrate that the basic residues play an important role in the interaction of the enzyme with COOH-f-DWCNT. In summary, our experimental and MD simulations results indicate that the conformation, dynamics, activity, and stability of the enzyme are affected by COOH-f-MWCNTs. In addition, the electrostatic, π-cation, and π-π stacking interactions are the main driving forces for the enzyme binding to the surface of COOH-f-MWCNTs. Consistent with the studies above, our results demonstrated that COOH-f-DWCNT has stronger affinity to the enzyme than CNT without the–COOH groups. For toxicity investigations, this research will provide an example of nanoscale toxicity resulting from direct interference with bio-macromolecules.

## Supporting information

S1 Fig TEM image of Multi-walled nanotubes (MWNTs)–COOH functionalized (20–30 nm, Purity >98%, -COOH). Carboxylation of MWCNTs is one of the widely used methods to improve the solubility in aqueous solution. COOH-f-MWCNTs were dispersed in ultrapure water for characterization study and in order to reduce agglomeration. COOH-f-MWCNTs Samples was sonicated several times intermittently (30s every 2 min) using a sonicator probe (Misonix-700 Q, USA) at 30°C. Ultraviolet- visible (UV–vis) spectra of COOH-f-MWCNTs were monitored using a Nanodrop 2000 spectrophotometer (Thermo Scientific™ Nanodrop™) equipped with a 10-mm quartz cell over the range 190–800 nm and the intensity and position of wavelength maximum was determined. COOH-f-MWCNTs did not absorb in UV-Vis region. In addition, the CNTs dispersions were analyzed using TEM to reveal the size of the individual CNTs. S1 Fig shows TEM image of COOH-f-MWCNTs dispersed in ultrapure water. It is evident, from the image that all the COOH-f-MWCNTs provide hollow and tubular shapes. Based on the TEM image of S1 Fig, it can be concluded that the COOH-f-MWCNTs dispersed in ultrapure water present diameter of about 20–30 nm.
(TIF)

S2 Fig Two different initial configurations of COOH-f-DWCNT used in the system 3 and system 4. (a) and (b) Depict the first and last snapshots of system 3, respectively. (c) and (d) represent the first and last snapshots of the system 4, respectively. (e) The COM distance between COOH-f-DWCNT and Loop1, Loop2, and Loop3 of the S1 pocket during the MD simulations. As can be seen, Loop1 is further from COOH-f-DWCNT than Loop2 and 3.
(TIF)

S3 Fig Snapshots of the models containing CNTs without carboxyl groups. (a), (c), (e), and (g) are the first snapshots in the Model 1, Model 2, Model 3, and Model 4, respectively. (b), (d), (f), and (h) are the last snapshots in the Model 1, Model 2, Model 3, and Model 4, respectively.
(TIF)

S4 Fig The DSSP analysis for (a) the system 3 and (b) the system 4.
(TIF)

S5 Fig (a) The number of hydrogen bonds between S1 pocket and -COOH groups of COOH-f-DWCNT in system 3 over the simulation time. (b) The number of contacts between COOH-f-DWCNT and the enzyme for all four systems during the MD simulation time.
(TIF)

S1 Table The binding energy (ΔG_binding_) of residues of S1 pocket with favorable *ΔG*_*binding*_ < -2 kJ.mol^-1^ in system 3. (PDF)

S2 Table The contribution energy of each basic residue to the total binding energy. The residues had absolute value of binding energy < -60 kJ.mol^−1^.
(PDF)

S3 Table The calculated energies for the systems containing CNT with (S1, S2, S3, and S4) and without (M1, M2, M3, and M4) the carboxyl groups. (PDF)
