# Supplementary figures and images for: The potential impact of carboxylic-functionalized multi-walled carbon nanotubes on trypsin: A Comprehensive spectroscopic and molecular dynamics simulation study
Source: PLoS One. 2018 Jun 1;13(6):e0198519. doi: 10.1371/journal.pone.0198519 (PMC5983559; doi:10.1371/journal.pone.0198519)

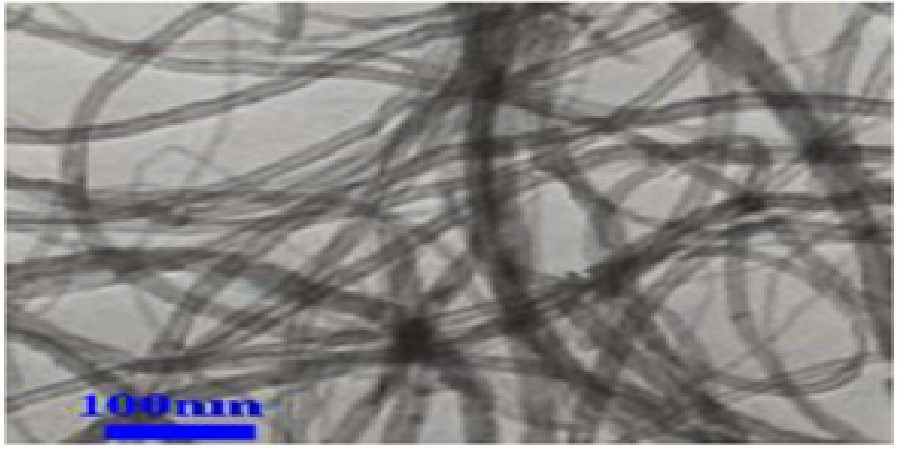

Supplement: S1 Fig — Carboxylation of MWCNTs is one of the widely used methods to improve the solubility in aqueous solution. COOH-f-MWCNTs were dispersed in ultrapure water for characterization study and in order to reduce agglomeration. COOH-f-MWCNTs Samples was sonicated several times intermittently (30s every 2 min) using a sonicator probe (Misonix-700 Q, USA) at 30°C. Ultraviolet- visible (UV–vis) spectra of COOH-f-MWCNTs were monitored using a Nanodrop 2000 spectrophotometer (Thermo Scientific™ Nanodrop™) equipped with a 10-mm quartz cell over the range 190–800 nm and the intensity and position of wavelength maximum was determined. COOH-f-MWCNTs did not absorb in UV-Vis region. In addition, the CNTs dispersions were analyzed using TEM to reveal the size of the individual CNTs. S1 Fig shows TEM image of COOH-f-MWCNTs dispersed in ultrapure water. It is evident, from the image that all the COOH-f-MWCNTs provide hollow and tubular shapes. Based on the TEM image of S1 Fig, it can be concluded that the COOH-f-MWCNTs dispersed in ultrapure water present diameter of about 20–30 nm. (TIF) [file pone.0198519.s001.tif]

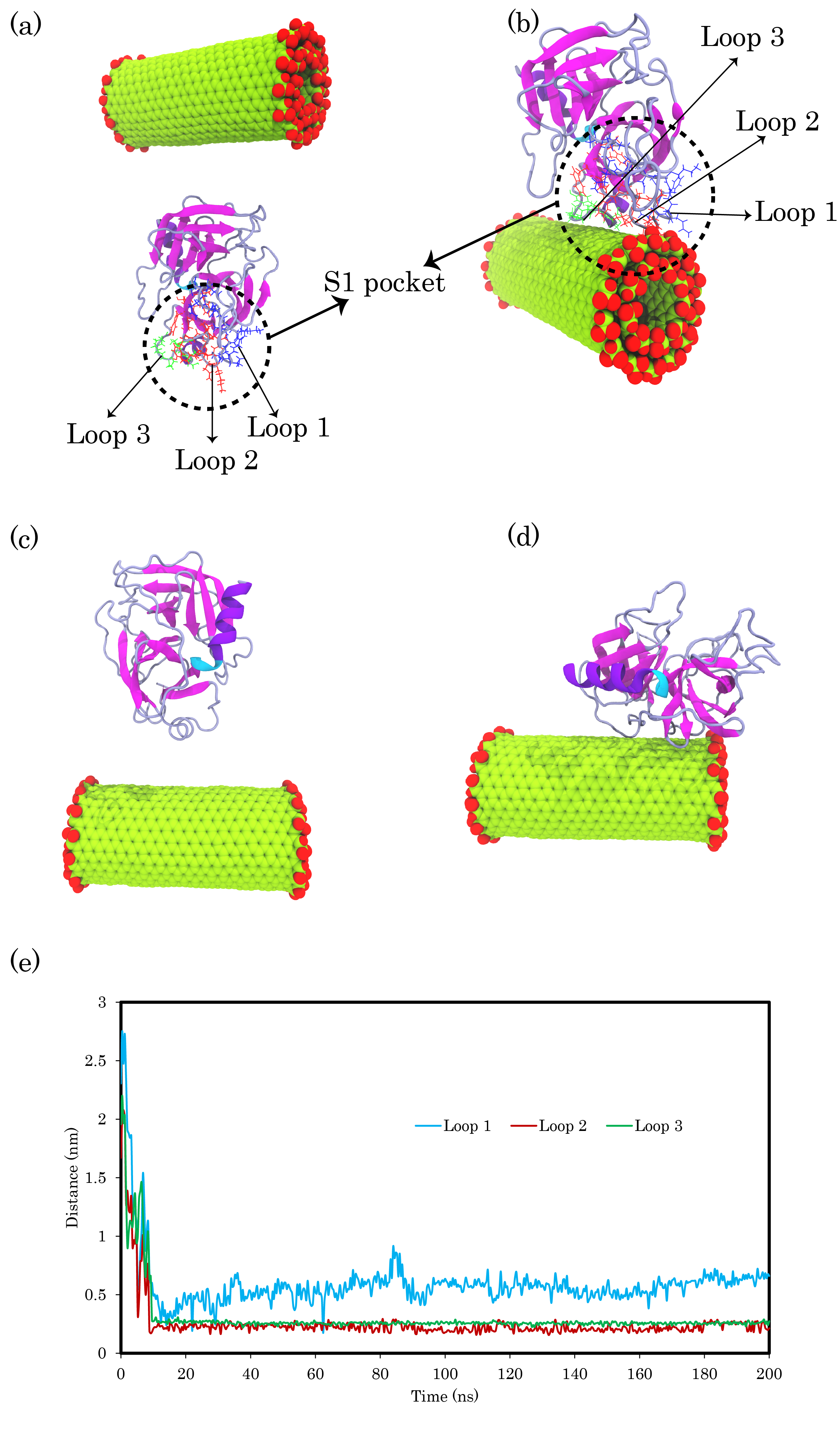

Supplement: S2 Fig — (a) and (b) Depict the first and last snapshots of system 3, respectively. (c) and (d) represent the first and last snapshots of the system 4, respectively. (e) The COM distance between COOH-f-DWCNT and Loop1, Loop2, and Loop3 of the S1 pocket during the MD simulations. As can be seen, Loop1 is further from COOH-f-DWCNT than Loop2 and 3. (TIF) [file pone.0198519.s002.tif]

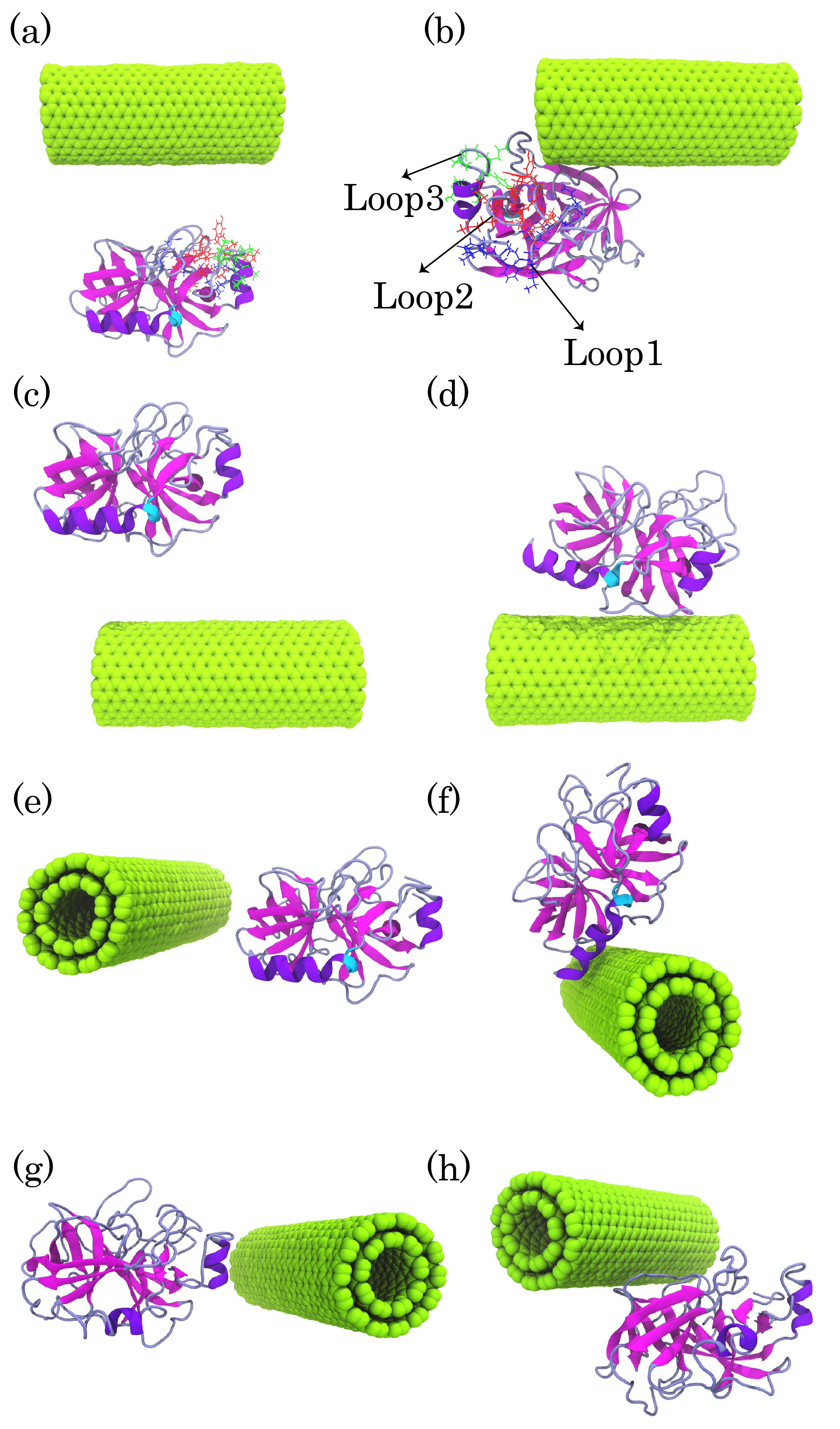

Supplement: S3 Fig — (a), (c), (e), and (g) are the first snapshots in the Model 1, Model 2, Model 3, and Model 4, respectively. (b), (d), (f), and (h) are the last snapshots in the Model 1, Model 2, Model 3, and Model 4, respectively. (TIF) [file pone.0198519.s003.tif]

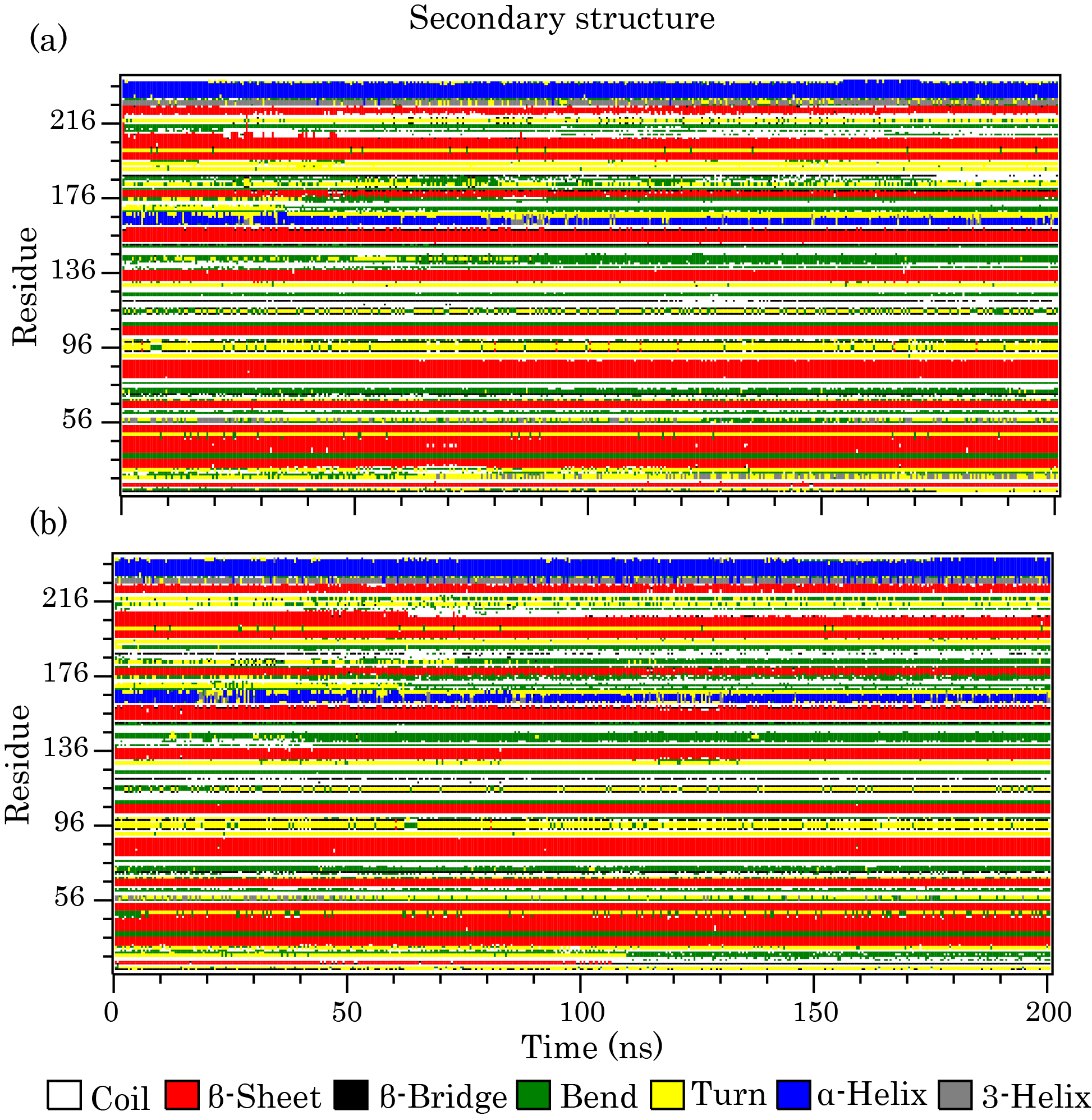

Supplement: S4 Fig — The DSSP analysis for (a) the system 3 and (b) the system 4. (TIF) [file pone.0198519.s004.tif]

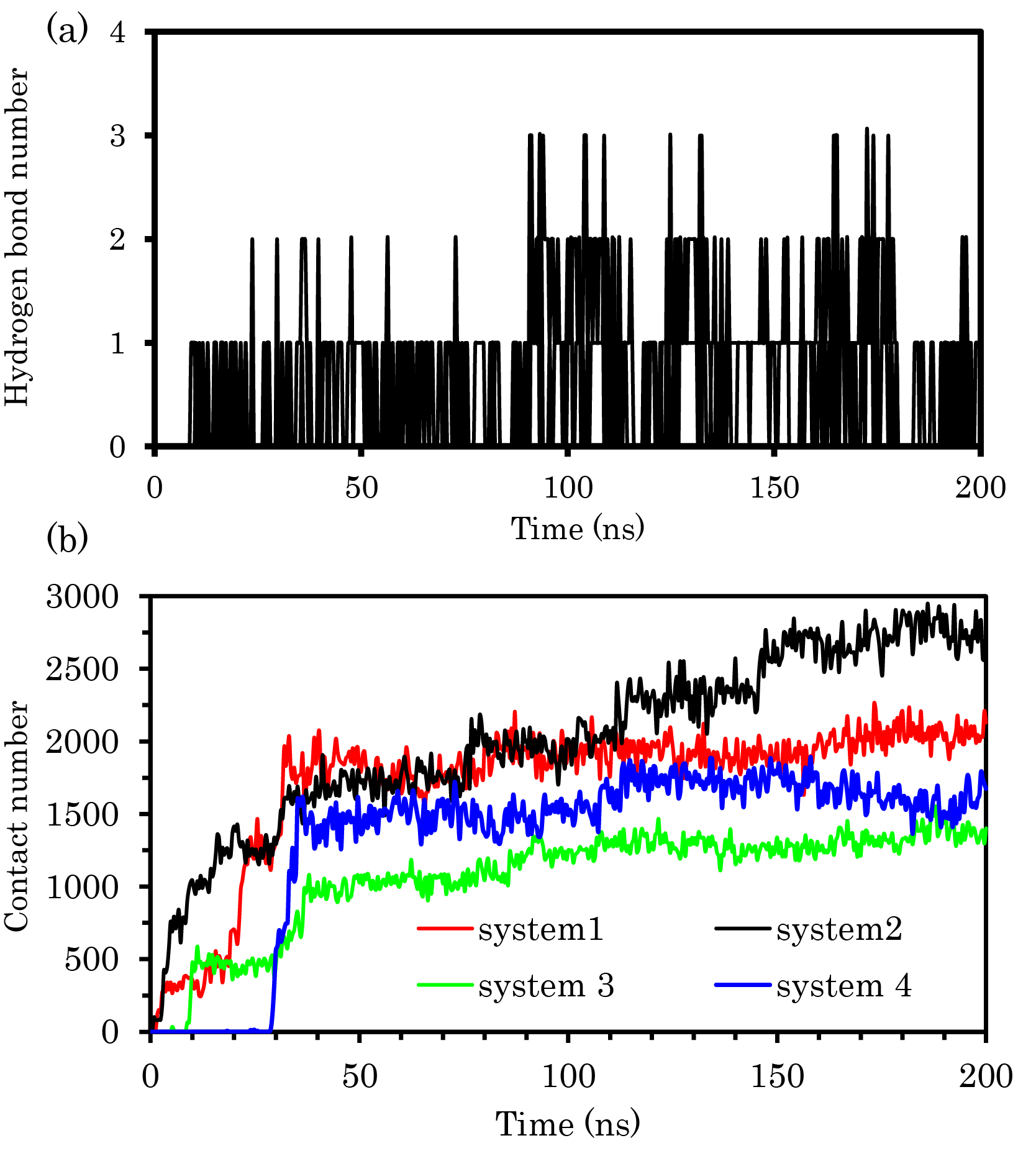

Supplement: S5 Fig — (a) The number of hydrogen bonds between S1 pocket and -COOH groups of COOH-f-DWCNT in system 3 over the simulation time. (b) The number of contacts between COOH-f-DWCNT and the enzyme for all four systems during the MD simulation time. (TIF) [file pone.0198519.s005.tif]
