# Supplementary material for: The potential impact of carboxylic-functionalized multi-walled carbon nanotubes on trypsin: A Comprehensive spectroscopic and molecular dynamics simulation study
Source: PLoS One. 2018 Jun 1;13(6):e0198519. doi: 10.1371/journal.pone.0198519 (PMC5983559; doi:10.1371/journal.pone.0198519)
Supplement: S1 Table — (PDF) [file pone.0198519.s006.pdf]

Table S1. The binding energy ($\Delta G_{binding}$) of residues of S1 pocket with favorable $\Delta G_{binding}$ < -2 $kJ.mol^{-1}$ in system 3.

Residue	$\Delta G_{binding}$
**LYS-222**	-70.4983
**LYS-224**	-65.4546
**LYS-169**	-61.0216
**GLN-175**	-2.1505
**LYS-188**	-63.4577
