# Supplementary material for: The potential impact of carboxylic-functionalized multi-walled carbon nanotubes on trypsin: A Comprehensive spectroscopic and molecular dynamics simulation study
Source: PLoS One. 2018 Jun 1;13(6):e0198519. doi: 10.1371/journal.pone.0198519 (PMC5983559; doi:10.1371/journal.pone.0198519)
Supplement: S2 Table — The residues had absolute value of binding energy < -60 kJ.mol−1. (PDF) [file pone.0198519.s007.pdf]

Table S2. The contribution energy of each basic residues to the total binding energy. The residues had absolute value of binding energy < -60 kJ.mol^{-1}.

System	Residue
**System 1**	Lys-60, Arg-62, Lys-145, Lys-222, Lys-224
**System 2**	Arg-117, Arg-125, Lys-159
**System 3**	Lys-169, Lys-222, Lys-224
**System 4**	Lys-159
