# Supplementary material for: The potential impact of carboxylic-functionalized multi-walled carbon nanotubes on trypsin: A Comprehensive spectroscopic and molecular dynamics simulation study
Source: PLoS One. 2018 Jun 1;13(6):e0198519. doi: 10.1371/journal.pone.0198519 (PMC5983559; doi:10.1371/journal.pone.0198519)
Supplement: S3 Table — (PDF) [file pone.0198519.s008.pdf]

Table S3. The calculated energies for the systems containing CNT with (S1, S2, S3, and S4) and without (M1, M2, M3, and M4) the carboxyl groups.

Energy (kJ/mol)	System							
	**S1**	**M1**	**S2**	**M2**	**S3**	**M3**	**S4**	**M4**
**Van der Waals energy**	-245.9	-160.2	-467.2	-308.2	-37.3	-380.5	-444.1	-21.3
**Electrostatic energy**	-1157	0	-1222.1	0	-731.1	0	-1481.3	0
**Non-polar binding energy**	-261.4	-170.8	-495.4	-325.2	-39.4	-403	-470.3	-22.7
**Polar binding energy**	-1034.1	0	-1095.3	0	-689.7	0	-1287.1	0
